# Supplementary material for: Are carbon emissions trading and green financial instruments synergistic? -Comprehensive quantitative research based on content analysis
Source: PLoS One. 2024 Mar 7;19(3):e0298601. doi: 10.1371/journal.pone.0298601 (PMC10919601; doi:10.1371/journal.pone.0298601)
Supplement: S1 Data — (ZIP) [file pone.0298601.s001.zip › Data/地级市Z_F工作报告.zip!/╜¡╬≈/╨┬╙α╩╨/└┤╫╘╣1⁄2╓┌║┼í╛┬φ┐╦╩2╛▌═°í┐_2013.docx]

各位代表：

　　现在，我代表市人民政府向大会报告政府工作，请予审议，并请各位市政协委员和其他列席会议的同志提出意见。

　　一、2012年工作回顾

　　2012年，是我市经济社会发展排难而进、奋发有为的一年。在省委、省政府和市委的正确领导下，全市上下深入贯彻落实科学发展观，牢牢把握稳中求进工作总基调，克服宏观经济环境不利影响，团结拼搏，开拓创新，全市经济社会实现平稳发展。预计（下同）全年实现生产总值840亿元，增长10%；财政总收入116.89亿元，增长5.1%，其中地方财政收入83.23亿元，增长19.9%；固定资产投资754.8亿元，增长20.5%；社会消费品零售总额155.4亿元，增长17%；引进内资423亿元，增长30%；城镇居民人均可支配收入22480元，农民人均纯收入10047元，均增长14%；居民消费价格涨幅控制在2.6%。

　　着力转变发展方式，工业经济平稳运行。转型升级步伐加快。大力实施“十百千亿”工程，新材料、动力与储能电池产业快速增长，江锂年产2万吨电解镍技改项目建成投产，江锂、赣锋锂业主要产品产量增长40%以上。电子信息产业加快发展，沃格光电、盛泰科技、好英王光电等企业产值翻番。赛维先后推出M2、M3新型高效多晶硅片，转换效率提高到17.5-18%。十大战略性新兴产业增加值占规模以上工业的比重达60.5%。园区新建标准厂房34.8万平方米。分宜工业园列入省级重点工业园。袁河经济开发区列入省级工业园区管理序列。非公经济主体数量增长12%，增加值占生产总值的比重达61%。招商引资成效明显。围绕“三项政策”和机械装备、动力与储能电池、户外照明、花卉苗木、文化旅游等产业，成功举办13场大型招商推介活动，签约引进亿元以上项目114个，其中新能源汽车等投资10亿元以上项目15个。对接央企招商取得新进展，中汽零、中电投、中国五矿等企业进驻，与航天万源、国电电力、中核集团等达成合作意向。帮扶企业渡过难关。把保企业稳生产作为中心，成立三大产业服务团队，派出干部500多名，挂点帮扶企业150多家，协调解决企业存在的困难和问题。实施“一企一策”，组建专门工作组，重点帮扶赛维等光伏企业应对困难。出台了《促进经济发展暂行办法》《关于促进小型微型企业发展的意见》等政策措施，兑现优惠政策资金2.46亿元，帮助企业融资178.7亿元。

　　着力实施项目带动，发展后劲持续增强。项目建设加快推进。新开工亿元以上项目115个，累计完成投资281.7亿元，其中工业项目48个，完成投资145.4亿元。100个重点项目全部在建，完成投资200亿元。成功争取蒙吉铁路途经新余，赣铁物流园等配套项目前期工作有序开展，杭南长客运专线（新余段）建设顺利推进，白梅水利枢纽工程列入国家大中型水库“十二五”建设规划。新电2台66万千瓦机组项目前期工作扎实推进，分电2台100万千瓦机组项目通过初可研。“三项政策”稳步实施。节能减排财政政策综合示范实施方案在全国8个试点城市中第一个获批，获批综合奖励项目6个、资金5.37亿元，获批现有政策项目13个、资金2.4亿元；“城市矿产”示范基地开工项目10个，新签约项目8个，获补助资金1.15亿元；资源枯竭城市转型规划通过省级初审，项目储备库收集八类项目262个，累计获转移支付资金5.03亿元。项目争取成效显著。建立常态调度机制，对接落实项目46个、资金170.4亿元。完成国家新能源示范城市规划，积极申报国家新能源示范城市。成功申报金太阳示范项目4个，总装机容量57兆瓦，获补助资金3.2亿元。列入省重点工程调度项目12个。争取省级用地指标6357亩。双林镇被列为第三批全国发展改革试点城镇。

　　着力夯实“三农”基础，现代农业稳步提升。兑现强农惠农政策补贴资金1.33亿元，争取现代农业等项目资金7782万元。农牧渔业全面增产，粮食生产实现“九连增”。农业产业化加快推进。出台《关于大力推进农业产业化发展的意见》，新增国家级龙头企业1家、省级14家；2家农民专业合作社获全国表彰，新增省级示范社8家。38家省级以上龙头企业完成销售收入104亿元，带动17.8万农户增收3.74亿元。特色农业发展壮大。制定了七大特色产业发展规划。果业投产面积达15万亩。新余蜜桔种植面积达12.75万亩，被列为全国特色柑桔品种优势产区，荣获“中国蜜桔之乡”称号。葡萄产区列为全省标准化生产科技示范区，早熟梨列入全省优势农产品区域规划。新增高产油茶2.3万亩、商品蔬菜基地8400亩。30万头生态养猪基地基本建成。基础设施日益完善。完成农田水利基本建设投资4.65亿元，创历年之最。31座小型病险水库除险加固工程、分宜县和渝水区小农水重点县项目年度建设任务全面完成。分宜县被列为国家现代农业示范区，渝水区被评为全国农田水利基本建设先进县（区）。建设高标准农田4万余亩，完成土地开发整理9.4万亩，新增耕地8780亩。新增农村自来水受益人口7.9万人。

　　着力抓好协同创新，科技研发取得突破。出台《关于进一步推动科技创新的若干意见》，市本级财政安排各类科技资金1969万元，全社会研发经费占生产总值比重达2.6%，“543211”科技创新工程全面完成，“六个一”工程建设获省政府表彰。创新平台建设加强。国家锂材料及应用高新技术产业化基地获批，国家光伏产品检测中心获准筹建，建立了新能源、金属新材料省级战略性新兴产业基地。企业创新能力提高。省级以上高新技术企业达15家、创新型企业12家。国家级科技项目立项12项，其中“863”计划4项；省级立项42项，获资金支持2000万元；开发省级重点新产品45项。产学研一体化加快。出台《促进产学研合作办法》，与国内外高校、科研机构共建研发机构12个，引进技术48项，专利授权32件。与中国地质大学签订全面合作协议，合作重点高校达7家。赛维院士工作站和博士后工作站、中国地质大学新余新能源材料研究院、华南理工大学新余工业技术研究院成立，国家新能源产品检测重点实验室落户。人才队伍不断壮大。引进创新创业高层次人才150余人。4人入选国务院、省政府特殊津贴人选。新增国家级技能大师工作室1个。2名外国专家获“庐山友谊奖”，1名获“国际科技合作奖”。荣获“新能源产业合作育人创新城市”称号。

　　着力推进统筹发展，城乡面貌焕然一新。统筹城乡发展总体实现程度78.27%，城镇化率65.58%。规划体系逐步完善。以市域规划为统领，基本实现中心城区和县区、部分中心镇控制性详细规划全覆盖，重点编制、修编了城市风貌、综合交通和中心城区停车场、商业网点、农贸市场、社区建设等专项规划。城市功能不断增强。“三城两区”建设累计完成投资278亿元。公园路等9条“断头路”基本打通，仙女湖大道西延段、新余大桥等建成通车，环城南路、钟灵大道、高铁站前广场交通枢纽等项目顺利推进。改造维修城区21条主干道路面7.1万平方米。虎头山？？园基本建成。主城区民用天然气置换全面完成。建设改造农贸市场3个。建成区绿地率、绿化覆盖率、人均公园绿地面积分别达51.96%、53.37%、18.45平方米。中心镇建设稳步推进。67个基础设施建设项目累计完成投资20亿元。11个中心镇集镇人口达11.37万人，增长9.8%。罗坊、双林两镇通过国家绿色低碳重点小城镇专家组评审考核。建设和谐秀美乡村精品示范点8个，在全省率先对辖区高速公路沿线181个村庄改造提升，推进了317个新农村建设点村庄整治，完成农村危房改造1680户。建设农村公路156.1公里。新建村级农民体育健身场所36个。城市管理水平不断提升。成立城市管理委员会，推行网格化管理，数字城管监督指挥系统投入运行，“大城管”新格局初步构建。成功创建省级文明城市，市容环境明显改善。

　　着力优化产业结构，现代服务业加快发展。第三产业实现增加值265亿元，增长11.4%；占生产总值比重31.6%，提高了4.2个百分点。现代物流业发展提速。赣西中心物流园配套设施加快建设，客运西站全面竣工，入驻物流项目12个。赣西万商红商贸物流中心等10个在建项目快速推进，完成投资28.8亿元。道路货物运力增长11%。商贸流通业强力推进。成功获批全国农产品现代流通综合试点城市、中小商贸流通企业服务体系建设试点城市。“1020工程”完成投资22.7亿元，实现销售收入63.2亿元。星辉农产品批发市场、新余国际商城、正大钢材市场等项目快速推进，广城建材家居博览中心等投入使用。金融业创新发展。各项融资余额1175亿元，增长17%。新余农商银行挂牌运营，孔目江成功村镇银行开业，新组建小额贷款公司2家、获准筹建1家，新引进保险公司分支机构3家。城投公司第二期企业债券成功发行。文化产业稳步发展。抱石文化创意园主体工程完工，天工文化创意产业园列入省级首批服务业集聚区。原创动画连续剧《天工开物—开心岛》获国家优秀动画片奖并在央视上映。旅游业快速发展。接待游客681.2万人次，增长24.1%；实现旅游总收入47.3亿元，增长41.3%。仙女湖景区总规修编完成，5A级景区创建启动。百丈峰养生度假区、中国洞村、仙女湖国际大酒店、锦湖宾馆等项目快速推进，佛教文化博览园一期工程投入使用。

　　着力改善人居环境，生态文明成果丰硕。环境保护力度加大。成为全国第一个合同环境服务试点地级市。廖家江、贯早江环境治理初见成效。新建污水收集管网15公里。全面取缔仙女湖网箱养鱼。完成造林11.13万亩，被评为全省造林绿化“一大四小”工程建设先进市。启动“森林城乡、绿色通道”建设，完成通道绿化提升107.7公里。仙女湖、孔目江两个主要饮用水源保护区水质稳定在Ⅱ类以上，袁惠渠、袁河水质基本达到Ⅲ类，城区空气质量基本达到二级标准。在全省率先编制生态文明规划，建成国家级生态村1个，省级生态乡镇17个、生态村20个。节能减排工作扎实推进。单位GDP能耗下降5.5%，主要污染物削减任务全面完成。高新区污水处理厂升级改造主体工程完工。碳排放权交易试点工作获批。在全省率先启动机动车“黄绿标”管理。国家节水型城市创建工作启动。重金属污染治理成效显著。种植苗木治理重金属污染土地的经验得到省委省政府充分肯定，被评为全省苗木发展先进市。赣西花木大世界、兰花博览园、凯光生态观光园等项目快速推进，新种植花卉苗木3.02万亩，总面积达6.23万亩。完成因污染严重而失去生存条件的4个自然村搬迁。矿山地质环境恢复治理规划编制启动。

　　着力改善民？？事业，社会保持和谐稳定。全市各级财政民生事业支出75.7亿元，全面或超额完成省政府下达的87项民生指标和市政府确定的10件实事。就业创业成效明显。新增城镇就业2.45万人，新增转移农村劳动力1.65万人。新发放小额贷款3.5亿元，增长90%，扶持和带动1.99万人就业创业。社会保障体系更加完善。在全省率先实现城乡居民养老保险全覆盖。城镇小集体企业职工参保工作全面完成。失地农民养老保险人数达4万人。开展社会保险“五险合一”统征分付试点。企业退休职工人均月增资195.89元。新农合年人均筹资标准提高到290元，参保率达98.5%。城乡低保月保障标准分别提高到350元、170元。农村“五保”集中供养、分散供养年标准分别提高到2640元、2160元。城镇“三无”特困群众供养标准月人均提高到400元。新（改）建农村敬老院13所，全省规模最大的民办养老机构银河园养老服务中心投入使用。发放高龄补贴2400余万元、物价补贴243.4万元。率先在全省建立重特大疾病救助制度。保障性住房建设有序推进。开工新建廉租住房1230套、公共租赁住房1620套，发放经济适用住房货币补贴134户、250.3万元，廉租住房租赁补贴5600户。完成棚户区改造24.5万平方米。各项社会事业全面发展。教育均衡发展初步实现，城乡义务教育各项免费、补助政策全面落实。率先在全省基本完成农村义务教育学校标准化建设。渝水区被评为全国“两基”工作先进区。建成渝水六小、仙女湖中学，北师大新余附属学校、分宜五中改扩建加快推进。落实学前三年行动计划，推行幼儿园分类管理。省职教园区路网建设基本成形，6所入园院校建设加快推进，新签入园院校2所。新余技师学院挂牌成立。基层卫生综合改革任务基本完成，在723个村卫生室实施了基本药物制度，分宜县县级公立医院综合改革有序推进。成立市食品安全委员会，食品安全工作获全省先进，两次代表全省接受国家检查获充分肯定。全民健身活动广泛开展，获省第四届全民健身运动会第三名，成功举办第七届市运会和第三届环鄱阳湖国际自行车（新余站）大赛。人口计生工作继续保持全省领先地位。实现全国双拥模范城“六连冠”。社会管理进一步加强。选派了72名群众工作特派员。所有乡镇（办）设立社会管理服务中心，实现矛盾纠纷化解常态化。创建了一批和谐社区和平安单位。第八届村（居）委会换届选举全面完成。公共安全体系和企业安全生产基础建设不断加强，安全生产形势稳定。应急救援装备全省领先。刑事案件发案率下降7.6%，赴省进京上访保持全省最低，没有发生影响稳定的群体性事件，全市社会政治稳定。

　　着力创新体制机制，政务环境不断优化。深化行政管理体制改革，全面落实机构编制实名制管理。深化部门预算改革，着手理顺市、县（区）事权关系。出台《关于支持促进新余高新区加快发展的意见》，推行大部门制运行、企业化运作模式。投融资体制改革稳步推进，组建了发展投资集团。制定《2013-2015年文化改革发展规划纲要》，荣获“全国文化体制改革工作先进地区”称号。中小学教师职称制度改革试点全面启动。城乡“一元化”户籍管理制度、农村产权制度、宅基地置换城镇住房、土地流转制度等改革有序推进，农村集体土地“三权”登记发证工作进展顺利。国有林场改革扎实有效。企业一套表统计改革稳步推进。集中整治“庸、懒、散”和“假、浮、蛮”，行政服务水平进一步提高。全面清理行政许可和非行政许可事项，实行行政审批事项网上办理。智慧新余建？？启动，政府数字化综合办公平台投入运行，入选中国城市信息化50强。坚持依法行政，行政复议委员会试点工作成效明显。加强廉政建设和反腐败工作，深化重点领域专项治理，切实解决损害群众利益的突出问题。审计、质监、海关、检验检疫、民族宗教、外事侨务、涉台事务、驻外机构、机关事务、防震减灾、地质找矿、方志档案、妇女儿童、科普、气象、人防、证券保险、邮政通信、烟草、盐业、海事、国防动员、民兵预备役、社科研究、红十字会人道援助和慈善事业、残疾人及老龄等工作取得新成绩。

　　各位代表，2012年，新余作为全省受宏观经济环境影响最大、范围最广、程度最深的地区，遇到的困难前所未有，经受的考验前所未有，能在如此困难的情况下取得这样的成绩，实属来之不易。这是我们按照中央和省委、省政府的决策部署，在市委的坚强领导下，在市人大、市政协的监督支持下，在社会各界的大力帮助下，全市各级政府依靠广大人民群众，凝心聚力、奋力拼搏的结果。在此，我代表市人民政府，向辛勤工作在全市各条战线上的广大干部群众，向人大代表、政协委员和离退休老同志，向各民主党派、工商联、无党派和社会各界人士，向中央和省驻市单位、来余投资企业，向驻市解放军、武警官兵和公安干警，表示衷心的感谢并致以崇高的敬意！

　　在肯定成绩的同时，我们也清醒认识到，全市经济社会发展还面临不少困难和问题：发展不足，结构不优，服务业发展相对滞后；受外部环境影响，部分企业生产成本上升、经营困难、效益下滑，在建大项目、好项目不多，新兴产业尚处在培育期，经济发展、产业转型面临较大压力；社会转型期矛盾凸显，公共服务和社会保障水平有待提高，加强和创新社会管理还有大量工作要做；少数机关干部依法行政能力、破解难题能力和执行力、创新力不强等问题还不同程度存在。对此，我们一定认真对待，切实加以解决。

　　二、2013年工作任务

　　2013年，是全面深入贯彻落实党的十八大精神的开局之年，是提前全面建成小康社会和实现城乡一体化的重要一年。政府工作的总体要求是：全面贯彻落实党的十八大和中央、省经济工作会议精神，坚持以邓小平理论、“三个代表”重要思想、科学发展观为指导，按照稳中求进的工作总基调，围绕建设和谐富裕文明新余的目标，提前全面建成小康社会和实现城乡一体化，以提高经济增长质量和效益为中心，进一步强化创新驱动，着力在解放思想、深化改革、调整结构、扩大投入、惠及民生、真抓实干上下功夫，实现经济平稳较快发展，促进社会和谐稳定。全市经济社会发展的主要预期目标是：生产总值增长11%、力争13%，财政总收入增长16%、力争20%，地方财政收入增长10%、力争15%，规模以上工业主营业务收入增长15%，固定资产投资增长20%，社会消费品零售总额增长15%，外贸出口总额增长5%、力争10%，实际利用外资增长10%、内资增长20%，城乡居民可支配收入增长13%。居民消费价格涨幅控制在3.5%以内，人口自然增长率控制在省定目标以内。

　　围绕上述目标，重点做好以下几个方面工作。

　　（一）推进转型升级，发展新型工业

　　以“积极融入鄱阳湖生态经济区、加快建设国家新能源科技城”为主线，全力推进新型工业化。

　　力促产业升级发展。大力实施“十百千亿”工程，加快经济转型和结构调整，着力构筑特色鲜明和竞争力强的新型工业体系。通过技术改造提高传统产业现代化水平，重点抓好新钢长材节能增效改造、江锂年产12万吨高纯氧化镁等循环经济、海螺年产100万吨矿渣微粉、钢丝厂异地搬迁技改等项目建设。大力培育战略性新兴产业，加快户外照明、医药食品产业园、正华电动汽车产业园、中投新能源锂聚合物动力电池生产线、中恒通汽车车桥生产线、江西风驰、中亚保温材料等项目建设，力促中汽零汽车零部件生产基地、广东光电等产业转移项目落户。全力抓好企业帮扶，强化领导联系企业帮扶工作机制。着力发展产业投资基金，支持重点行业、企业做大做强。抓住国家促进光伏产业健康发展的政策机遇，帮促光伏企业尽快恢复生产，引导企业发展光伏电站和智能微电网终端应用产品。

　　加快三大园区建设。充分发挥园区主战场作用，提高单位面积投资强度，盘活闲置土地和厂房，新建标准厂房100万平方米。高新区重点推进新能源等六大特色产业园和三大投融资公司建设。分宜工业园重点推进基础设施建设，打造动力与储能电池和新材料特色产业园。袁河经济开发区重点对接新钢、盘活存量、整合资源，打造特钢和机械装备产业园。

　　推动小微企业发展。全面落实促进小微企业发展的各项政策，放宽市场准入条件，加大财税扶持力度，抓好中小企业公共服务平台和孵化中心建设。增强中小企业信用担保实力，创新金融产品，满足小微企业贷款短、频、快需求。出台鼓励创新创业政策，支持民间资本进入政府投资及公共服务领域，加强创业培训，推动全民创业，支持本土企业加快发展。

　　（二）推进统筹发展，加快城乡一体化

　　致力建设赣西区域中心城市，全力推进新型城镇化。

　　强化规划引领。加快主城区控制性详细规划编制，修编中心城区排水专项规划，启动老城区、城企和城乡结合部改造专项规划编制，推进中心镇控制性详细规划及村庄规划编制、修编。维护规划权威，完善实施机制，强化实施监管。

　　完善城区功能。实现城市建设由外延扩张向内涵提升、功能完善、综合承载力提高转变，城镇化率达68%。计划安排城建项目32个，年度投资13.3亿元。实施城区道路畅通工程，完善交通路网体系。建成文化中心、钟灵大道、高铁站前广场交通枢纽等工程，抓好市民服务中心建设。推进袁河生态新城整体开发，加快湖河一体化规划实施。加快雨污管网建设，力争污水处理集中处置率达96.89%。推进公园游园、主干道景观提升改造，新增绿地面积3万平方米，建成区绿地率、绿化覆盖率、人均公园绿地面积分别达52.46%、54.07%、18.95平方米。

　　加强城市管理。深入推进智慧新余建设，充分发挥城市管理委员会引领作用，抓好城市管理制度和责任体系落实，全面实施精细化、网格化管理，确保“数字城管”高效运行。完善地下管线档案统一管理，确保地下管线安全有序。加强和规范物业管理。

　　抓好中心镇建设。落实中心镇产业发展规划，逐步形成“一乡一产业，一村一特色”发展格局，以特色主导产业支撑中心镇可持续发展。创新融资担保机制，拓宽资金筹集渠道，广泛吸纳社会资金参与中心镇建设。抓好自来水供给、污水处理、垃圾分类收运处理等设施建设，推进公共服务均等化，确保集镇人口新增8%。

　　完善城乡公路体系。围绕打造主城区至周边县市“一小时经济圈”和各乡镇间“半小时经济圈”，加快余新货运快速公路建设，确保樟排线（罗坊至水西段）一级公路改建主体工程、河良线江口危桥改造工程完工，做好洞宜线改造前期准？？？。

　　建设和谐秀美乡村。抓好180个新农村点建设，打造主要交通沿线精品示范线和10个精品示范村。抓好30个“一村一品”项目建设，培植一批特色主导产业超过总产值70%的村庄。推进百万树木进村入户工程，完成新农村建设点植树20万株。加大农村新建住房新房型推行力度。深入实施农村清洁工程。积极申报省级以上历史文化名村和人居环境范例奖。

　　加快发展现代农业。稳定粮食播面150万亩，确保大宗农产品有效供给。推进农业产业化“1010工程”，力争新增省级以上龙头企业3家。抓好分宜县国家现代农业示范区、孔目江国家现代农业科技园、高新区省级现代畜牧产业园、市农机产业园、渝水区生态农业示范园等五大园区建设。推进特色农业发展，新增新余蜜桔1万亩、翠冠梨0.5万亩、高产油茶2万亩。着力打造仙女湖有机鱼品牌。推进渝水区界水乡北山1000亩20兆瓦光伏农业示范基地建设。实施农民专业合作社“十百千工程”，重点建设省级示范社10家、市级示范社100家，力争2015年全市农民专业合作社发展到1000家。抓好53座小（2）型病险水库除险加固、袁惠渠续建配套与节水改造项目建设，开工3座病险水闸除险加固工程。抓好渝水区第四批小农水重点县、中小河流治理重点县综合整治试点项目建设。完成永久基本农田划定，推进农村土地整治，完成造地4000亩，治理水土流失3万亩，建设高标准农田3万亩。

　　（三）推进结构优化，壮大第三产业

　　坚持以规划为龙头，以重点项目和龙头企业为抓手，建立发展引导资金和产业发展基金，全力推进现代服务业发展。

　　加速发展生产性服务业。大力发展现代金融业。引导金融机构聚集发展，加快商业银行机构及村镇银行引进步伐，力促新余农商银行达到银监会监管二级标准，助推孔目江成功村镇银行加快发展。新组建注册资本超过2亿元的小额贷款公司2家。力争高新区纳入“新三板”扩容范围。整合规范担保行业，加强社会信用体系建设。争取年末各项融资余额突破1240亿元，其中贷款余额突破850亿元，增长13.3%。大力发展现代物流业。出台扶持物流产业加快发展的专项政策，建立物流业发展的推进机制，重点抓好赣西中心物流园区和高新区、分宜县物流中心建设，推动物流龙头企业加快发展。整合信息化资源，加强与全国物流公共信息平台对接，促进铁路、水运、公路和航空等运输方式互连互通，构建统一物流口岸信息平台。

　　提升发展消费性服务业。大力发展商贸流通业。抓好商贸流通“1020工程”，重点支持赣西万商红商贸物流中心、东方巴黎家居广场、广城建材家居博览中心、新余国际商城和腾达电器、沃尔玛、步步高、洪客隆等商业企业做大做强。推进农产品现代流通综合试点，重点建设鲜活农产品、优质农产品、“放心粮油”、农资四大批发市场。大力发展休闲旅游业，实现旅游收入增长50%以上。推动仙女湖5A级景区创建，加快佛教文化博览园等项目建设，确保中国洞村景区建成开放。积极培育特色导游队伍，大力开发具有新余特色的旅游工艺品、纪念品。加快发展休闲农业和乡村旅游，新增休闲农业企业40家、农家乐80家。繁荣大众餐饮市场，加快星级酒店建设，开展旅游“名菜、名品、名店”系列创评。

　　大力发展新兴服务业。科学编制文化产业发展规划，深度挖掘抱石文化、天工文化内涵，把广播电视台、报社、抱石文化创意园等培育成文化产业的龙头。培育汽车文化产业。大力发展电子商务，着力建设大型骨干企业电子商务应用平台、中小企业电子商务服务平台和政府采购平台，支持兴邦产业、金利达、腾达电器等企业拓展电子商务领域。加强社区公共管理和公共服务平台建设，培育发展家政服务、养老服务、社区服务和家庭教育等新兴业态。培育发展中介服务市场。

　　（四）推进项目建设，扩大有效投入

　　坚定不移实施重大项目带动战略，以项目保投资、带产业、促发展。

　　加快重大项目建设。安排投资2000万元以上项目212个，总投资687亿元，年度投资280亿元。安排市重点项目100个，总投资491亿元，年度投资122亿元，其中新建项目50个，工业项目39个。全力抓好事关新余长远发展的重大项目，保障杭南长客运专线（新余段）建设，做好蒙吉铁路（新余段）开工准备，抓好洋坊站（新余西站）、良山站、赣铁物流园等项目选址和前期各项工作，推进煤储基地立项，争取新电2台66万千瓦机组立项和2台100万千瓦机组完成预可研、分电2台100万千瓦机组完成可研。充分利用国家“三项政策”，加大资金争取和项目推进力度，带动节能减排产业发展壮大。力争国家新能源示范城市、分布式光伏发电规模化应用示范区和城区老工业区整体搬迁改造项目获批。

　　完善项目推进机制。建立项目分级负责制，实行一个项目一名领导负责。推行招商引资项目合同预审和行政审批代理制，实施重大项目联审和限时办结制，提高行政审批效率和服务水平。完善重大项目进度通报、督查考核、责任追究制度，确保上半年签约项目年内全部开工、下半年签约项目年底前开工一半以上。严厉打击强装强卸、强揽工程行为，营造亲商、安商、护商环境。

　　提高项目招引实效。围绕延伸钢铁、新能源、新材料产业链，着力抓好新能源汽车、节能环保、机械装备、电子信息、户外照明、生物医药、绿色食品、花卉苗木和现代服务业等重点产业招商。调整招商重点，既主攻带动效果好的重大项目、高科技项目，也大力引进实实在在的符合产业政策的中小项目。强化县区招商主体作用，完善招商小分队管理、考核、培训和激励机制，重点充实工业园区专业招商力量。强化对接央企招商，抓好与国机集团、航天万源、国电电力、中国五矿等央企的对接合作。鼓励企业增资扩股。加快培育一批出口创汇能力强的骨干企业，建成新余口岸作业区海关监管场所。

　　（五）推进科技引领，提升创新能力

　　坚定不移实施科技兴市和人才强市战略，充分发挥科技第一生产力和人才第一资源作用，不断增强核心竞争力。

　　增强企业创新能力。突出企业创新主体地位，不断提高原始创新、集成创新和引进消化吸收再创新能力。支持企业建立研发中心和技术开发部，帮促江锂、赣锋锂业、江西华电等企业创建省级研发机构或博士后工作站，力促永盛矿冶、中冶南方、江西钢丝厂通过省级企业技术中心认定，新批省级以上高新技术企业5家、创新型企业3家、新产品30个。实施重大科技专项与科技示范工程，支持企业加快关键技术攻关、承担重大科技项目。加强市农科所等科研机构建设。实施知识产权优强企业培育工程，专利申请量、授权量均增长15%以上。

　　推进产学研一体化。加快运用高新技术、信息技术改造钢铁、化工、纺织等传统产业，突破和掌握一批核心技术，确保战略性新兴产业总产值进入全省设区市前三位，高新技术产业增加值占工业增加值的比重达30%，科技进步贡献率达53.2%。提升国家光伏工程技术研究中心等平台科技创新能力，？？设国家新能源产品检测重点实验室和国家光伏质检中心。深化与高校、科研机构的合作，争取更多科研成果在我市孵化。

　　加强人才队伍建设。落实人才发展规划，深化人才管理改革试验区建设。完善并出台《引进高层次人才实施意见》，全面落实人才引进和培养政策。实施专业技术人才知识更新工程、高技能人才振兴工程，培育创新创业领军人才、城市营销人才、金融专业人才和职业经理人队伍。

　　（六）推进重点改革，增强发展活力

　　在更大空间和更深层次上加快改革，最大限度激发社会创新、创造、创业活力，提高发展竞争力。

　　深化行政体制改革。健全部门职责体系，稳步推进大部门制改革。严格控制机构编制，全面清理和规范管理机关临时工作人员。加强政府债务风险防范，加强融资平台建设和发展，推行“融、用、管、还”管理模式。深化投资体制改革，积极推行政府投资项目代建制，完善项目预算、决算和审计机制。完善社会投资项目核准备案制度，减少社会投资项目核准事项，进一步放开社会投资领域。稳步推进事业单位分类改革和绩效工资改革。理顺市与县区事权管理体制。支持分宜工业园、袁河经济开发区尽快推广高新区企业化运作模式。

　　深化医药卫生改革。健全全民医保体系，推行按病种付费、总额预付等支付方式改革。深化基层卫生机构综合改革，健全多渠道补偿和绩效考核机制；巩固基本药物制度改革成果，推进基层卫生机构标准化建设和一体化管理。抓好分宜县县级公立医院综合改革。鼓励和支持社会办医，提高非公医疗机构床位数和服务水平。

　　深化农村各项改革。深入推进扩权强镇改革，推进市直管镇试点，强化中心镇管理功能。深化分宜县“一元化”户籍制度改革。推进国有林场改革，建设公益型林场4个、商品型林场1个；深化林权制度配套改革，稳步推进林权抵押贷款。深化农村产权制度改革，探索建立进城农民承包地、林地、宅基地等退出和补偿机制，实现宅基地换房1000户以上。推进农村土地承包经营权规范化流转，建立县、乡、村三级服务平台，中心镇土地流转率达20%，规范性流转合同签订率达40%。

　　（七）推进生态文明，建设美丽新余

　　以“建设全国节能减排财政政策综合示范城市和全国生态文明城市”为主线，把生态文明建设融入经济社会发展全过程。

　　强化节能减排。严格执行节能减排目标责任制，督导重点企业落实节能减排目标，单位GDP能耗下降3.5%，完成省政府下达的主要污染物减排任务。鼓励社会各界举报偷排、超排行为，加大违规排放处罚力度。落实节能减排财政政策综合示范实施方案，完善配套政策，抓好袁河绿色低碳工业平台、市碳排放权交易平台、能源监测管理中心、智能微电网、螺杆膨胀动力机推广、LED高效照明产品推广等6个典型示范项目实施；确保国家“城市矿产”示范基地13个项目建成投产；抓紧出台加快推进资源枯竭城市转型与可持续发展实施意见。积极推进合同能源管理。加快创建国家节水型城市。

　　发展低碳经济。大力发展循环经济，提高资源能源产出效率。实施30兆瓦太阳能光电项目和建筑面积150万平方米光（地）热应用项目，光伏发电总装机容量达70兆瓦；推进10万平方米既有建筑节能改造，新建绿色建筑20万平方米。扩大天然气利用规模，加快公交车、出租车、公务用车和公共场所天然气应用步伐，逐步开拓农村用气市场。

　　保护生态环境。抓好全国合同？？境服务试点，重点在环境治理、环保咨询、环境监测、环保产业等方面开展合同服务，推进新钢烧结脱硫、袁河工业平台污水处理、下村及良山污水处理厂改造、城市生活垃圾发电项目实施。确保仙女湖、孔目江两个主要饮用水源保护区水质稳定在Ⅱ类以上，城区空气质量稳定在国家二级标准。重点推进重金属污染治理和生态修复，新种花卉苗木10万亩。巩固国家森林城市建设成果，完成造林8万亩。全面推进“森林城乡、绿色通道”建设，抓好仙女湖大道西延段和钟灵大道等道路绿化，打造毓秀山、仙女湖、百丈峰等重点景区公路绿化精品工程。推进森林乡镇和森林村庄创建，确保全市80%以上乡镇成为省级生态乡镇。

　　（八）推进民生改善，发展社会事业

　　以“实现人均期望寿命80岁”目标为主线，把保障和改善民生放在更加突出的位置，让人民群众过上更好的生活。

　　为民办好实事。围绕解决好人民群众最关心最直接最现实的利益问题，突出扩大就业促进创业、完善社会保障体系、提高社会救助水平、强化医药卫生保障、促进教育公平和均衡发展、发展公益文化、加快保障性住房建设、改善居民出行条件、改善群众生产生活条件等方面，办好50件实事。

　　加强扶贫开发。统筹安排扶贫资金，探索建立扶贫开发多元化投入机制，构筑专项扶贫、行业扶贫、社会扶贫“三位一体”的大扶贫格局。加快产业扶贫，推进科技扶贫示范基地建设，拓宽贫困人口增收渠道，提升扶持对象自我发展能力。

　　发展社会事业。坚持教育优先发展战略。推进义务教育均衡发展，加快城区教育资源布局规划和建设；推进职业教育集约发展，整合职教资源，力争引进2所以上市外职业院校入驻职教园区，支持渝州科技职业学院升本，为我市产业发展提供技能型人才；推进高等教育内涵式发展，支持新余学院更名为江西工业学院，努力提升新余学院服务地方经济发展的能力。推进文化设施建设，强化文化市场监管，申报国家公共文化服务体系示范区。积极创建国家卫生城市，巩固基本药物制度，健全农村三级医疗卫生服务网络和城市社区卫生服务体系，推进城市公立医院内涵建设，提升卫生服务能力和水平。强化食品药品安全监管，确保不发生重大食品药品安全事故。打造群众体育赛事品牌，盘活市体育中心闲置资产，创出体教结合典型经验。抓好精神文明建设，提高市民素质，以创建全国文明城市引领各项创建活动。促进人口长期均衡发展，确保人口计生工作继续在全省领先。关心支持妇儿、老龄事业，发展残疾人事业和慈善事业。加强国防后备力量和人民防空建设，推进“双拥”和优抚安置。抓好全国第三次经济普查。做好新形势下的科普、气象、史志、外事侨务、民族宗教、驻外机构、防震减灾、邮政通讯等工作。

　　创新社会管理。完善党委领导、政府负责、社会协同、公众参与、法治保障的社会管理体制，加快形成源头治理、动态管理、应急处置相结合的社会管理机制，提高社会管理和服务科学化水平。完善社区组织管理体系，落实社区规划，推行新型社区服务管理模式，探索社会组织管理机制创新。完善社会治安综合治理，加强国家安全和人民防线工作，防范和打击各种违法犯罪，确保社会政治稳定。落实安全生产责任制和事故责任追究制，抓好重点行业、重点领域安全生产专项整治，坚决遏制重特大安全事故发生。全面推进依法治市，深入开展“六五”普法宣传教育，增强市民法制意识，加大法律援助？？度。坚持领导干部接访制度，高度重视群众来信来访，畅通规范群众利益诉求表达渠道，加强矛盾排查、调处化解，妥善处置各类群体性上访。

　　三、加强政府自身建设

　　切实转变机关作风，提高施政水平和行政效率，努力建设人民满意的政府。

　　建设法治政府。认真贯彻落实市委决策部署，积极发挥人大选举民主、政协协商民主作用，自觉接受人大法律监督、工作监督和政协民主监督，高质量办理人大议案、政协提案及其意见建议。认真听取各民主党派、工商联、无党派人士及各人民团体的意见，主动接受社会公众和新闻舆论监督，广开渠道听取群众意见建议。巩固行政复议委员会试点成果，落实依法行政各项制度。坚持公众参与、专家论证和政府决策相结合，完善重大决策社会稳定风险评估机制。深入推进政务公开，实现政务公开全覆盖。整顿财税秩序，支持财税部门依法征管。

　　建设效能政府。严格落实中央“八项规定”，静下心来谋发展，扑下身子抓落实，确保事事有着落、件件有回音。创新政府工作流程，推行扁平化管理，推进电子政务建设，全面推进政府数字化综合办公，提高工作效率和应急管理水平。完善绩效评价体系，严格目标管理，加强督查督办，从严整治不作为、乱作为，确保政令畅通。

　　建设廉洁政府。严格落实党风廉政建设责任制，健全完善惩治和预防腐败体系，全面推进公共权力规范运行预警机制建设。切实抓好电子政务统一监察平台建设全省试点工作。加强行政监察和财政、审计监督，做到绩效有评估、过失必追究。坚持分级负责、花钱预算、勤俭节约，集中财力办大事，把有限资金和资源用在发展经济、改善民生上。严格控制各项支出尤其是“三公”经费支出，逐步推行“三公”经费公开。

　　各位代表，新的一年，新余发展的任务非常繁重，我们深感责任重大。全市人民的信任，是我们做好工作的强大动力。让我们紧密团结在以习近平同志为总书记的党中央周围，在省委、省政府和市委的领导下，解放思想，改革开放，凝聚力量，攻坚克难，为建设和谐富裕文明新余，提前全面建成小康社会和实现城乡一体化而努力奋斗！
